# Morphological and Structural Characterization of (Pt, Au, and Ag) Nanoparticle/Zn-MOF-74 Composites

**DOI:** 10.1021/acsomega.3c09973

**Published:** 2024-05-10

**Authors:** Juliana Assunção Pereira de Figueiredo, Maximiliano Jesús Moreno Zapata, Laíse Serra Amorim, João Alves de Oliveira Neto, Douglas Rodrigues Miquita, Edmar Avellar Soares, Karla Balzuweit, Carlos Basílio Pinheiro

**Affiliations:** † Physics Department, Universidade Federal de Minas Gerais, Belo Horizonte 31270-901, Brazil; ‡ Centro de Microscopia, Universidade Federal de Minas Gerais, Belo Horizonte 31270-901, Brazil

## Abstract

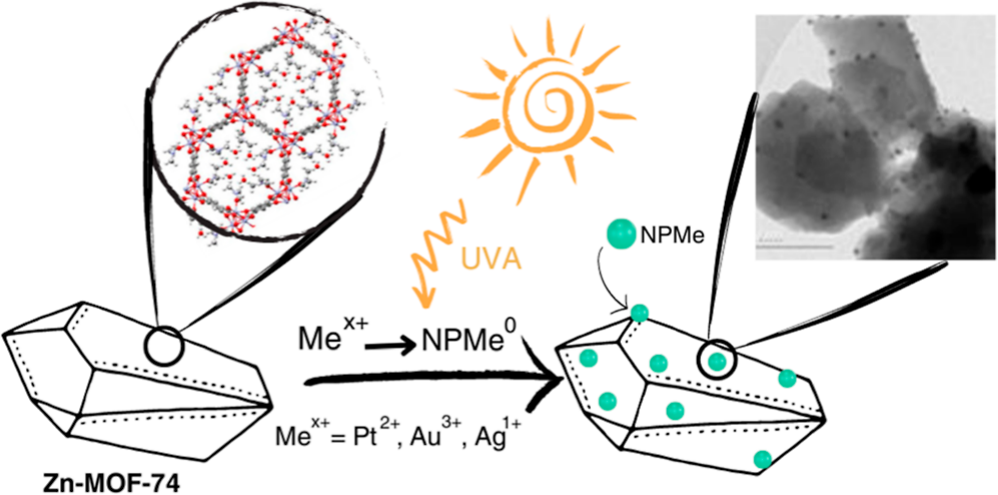

Metallic nanoparticles (NPs) were decorated onto Zn-MOF-74 crystals by photoreducing different metal precursors (Pt, Au, and Ag) using ultraviolet (UV) light in an aqueous solution with different metal concentrations without using additional stabilizers. X-ray diffraction revealed the three-dimensional structural integrity and crystallinity conservation of Zn-MOF-74 crystals during the UV decoration process. Raman spectroscopy showed a minor rearrangement in the structure of the Zn-MOF-74 crystal surface after NP decoration. X-ray photoelectron spectroscopy confirmed the metal oxidation states of Zn and NPs. High-resolution transmission electron microscopy images proved the surface decoration of Zn-MOF-74 crystals with spherical metallic NPs with diameters between 2.4 and 9.8 nm.

## 1 Introduction

Metal–organic frameworks (MOFs) are a broad class of crystalline materials composed of metal ions or metallic clusters interconnected by organic linkers to form porous networks.^1,2^ Owing to their structural and topological characteristics, such as large surface areas, tunable pore sizes, and unsaturated metal coordination sites, MOFs have applications across various fields, including adsorption,^3^ gas separation^4,5^ and storage,^5,6^ catalysis,^6,7^ magnetism,^8^ photoluminescence,^9^ chemical sensing,^10^ and biology.^11^ Among the known structures of MOFs, MOF-74 stands out due to its unique structural properties. MOF-74 is composed of a cluster of different divalent metals (Fe^2+^, Co^2+^, Zn^2+^, Cu^2+^, Ni^2+^, and Mn^2+^) and the 2,5-dihydroxyterephthalic (C_8_H_6_O_6_) ligand, resulting in a stable structure with one-dimensional infinite hexagonal open pores.^12^ MOF-74 can be synthesized via different synthetic routes, including the one-pot solvothermal method,^12^ microwave-assisted synthesis,^13^ vapor-assisted crystallization,^14^ sonochemical method,^15^ and room-temperature acid synthesis.^16^ Each method affords MOF-74 crystals with different sizes and morphologies while maintaining the same three-dimensional (3D) porous structure. The open porosity and stability of MOF-74 facilitate pore-emptying processes and allow molecules access to pores so they can actively interact with the active metallic sites or organic ligands of MOF-74.^17^ Furthermore, the structural stability of MOF-74, caused by strong interactions between metallic sites and organic ligands, ensures that pore-emptying/filling processes can be reversed without affecting its crystal structure.^18^ Owing to these characteristics, MOF-74 is a promising candidate material for green and sustainable technologies such as CO_2_ sorption and separation,^19^ molecule immobilization,^20^ and (bio)catalytic processes.^19,21^

Systems composed of Pt, Au, and Ag metal nanoparticles (NPs) supported on MOFs (henceforth called NPs/MOF) have attracted increasing attention from the scientific community because they combine the unique catalytic properties of metallic NPs with the physical and chemical selectivity of open-pore MOFs.^20,22,23^ The research in this field has focused on controlling the size, composition, nature of the dispersion, spatial distribution, and confinement of incorporated NPs within or over MOF matrices. Several NPs/MOF systems are used in catalytic assays, among which the most common are core–shell systems composed of MOFs and metallic NPs,^24−27^ NP-encapsulated systems,^28^ and NP-decorated MOF systems.^27,29^ In all these NPs/MOF systems, metals, which make up the secondary building unit (SBU) of MOFs, behave as selective interaction centers; the pore size of the MOF determines the geometrical properties of the host within cavities, and metallic NPs decorated onto MOFs afford additional electronic and catalytic properties to the NPs/MOF complexes.^25,27^

MOFs and NPs composites with core–shell structure (NPs@MOF) as well as NPs/MOF composites^30,31^ have also been synthesized to investigate whether the integration of MOF with plasmonic nanostructures enhances the unobserved properties of each component.^32^ In general, NPs/MOF composites have been obtained by reducing metallic cations in solution in the presence of MOF crystals, affording metallic NPs impregnated on the MOF surface.^33^ However, in many reported NPs/MOFs, a stabilizer is often required to control the shape of the deposited NPs. However, McGilvray et al.^34^ reported the synthesis of AuNPs using a photoreduction process, which afforded protected and unprotected NPs. They also found that the size of the synthesized NPs was related to the intensity of ultraviolet (UV) light used in the reduction process. Lollmahomed et al.^35^ demonstrated that this photoreduction process could be used to decorate metallic NPs on functionalized carbon nanotubes, ensuring the coverage and good aggregation of NPs on the MOF surface.

This study reports a straightforward and robust approach for synthesizing composites of Pt, Au, and AgNPs and Zn-MOF-74 (henceforth called MeNPs/Zn-MOF-74, where Me = Pt, Au, and Ag) using a postsynthetic process. In the designed method, Zn-MOF-74 crystals are synthesized via the standard solvothermal reaction^12^ and Pt, Au, and Ag NPs are produced and incorporated onto the Zn-MOF-74 crystal surface by photoreducing a metal precursor in a nondestructive environment for the crystals. The resulting MeNPs/Zn-MOF-74 (Me = Pt, Au, and Ag) composites were characterized via powder X-ray diffraction (PXRD), Raman spectroscopy, scanning electron microscopy (SEM), energy-dispersive X-ray spectroscopy (EDS), X-ray photoelectron spectroscopy (XPS), and transmission electron microscopy (TEM). Characterization results prove that the concentration of the metal precursor alone can be used to control the average size of NPs decorated onto the Zn-MOF-74 surface. These results provide new insights into the easy and inexpensive synthesis of NPs/MOFs for composites and core–shell nanostructure applications.

## 2 Experimental Section

### 2.1 Materials

2,5-Dihydroxyterephthalic acid (98 wt %), zinc nitrate hexahydrate [Zn(NO_3_)_2_·6H_2_O, 98%], *N*, *N*-dimethylformamide (DMF; 99.8%), platinum(II) acetylacetonate [Pt(acac)_2_, 99.99%], chloroauric acid solution (HAuCl_4_, 0.1M), silver nitrate (AgNO_3_, 99%), and Irgacure 2959^1^ (I-2959) used for the synthesis of Zn-MOF-74 and Pt, Au, and Ag NPs were of analytical grade and purchased from commercial suppliers.

### 2.2 Synthesis

#### 2.2.1 Zn-MOF-74 Synthesis

**Figure 1:**
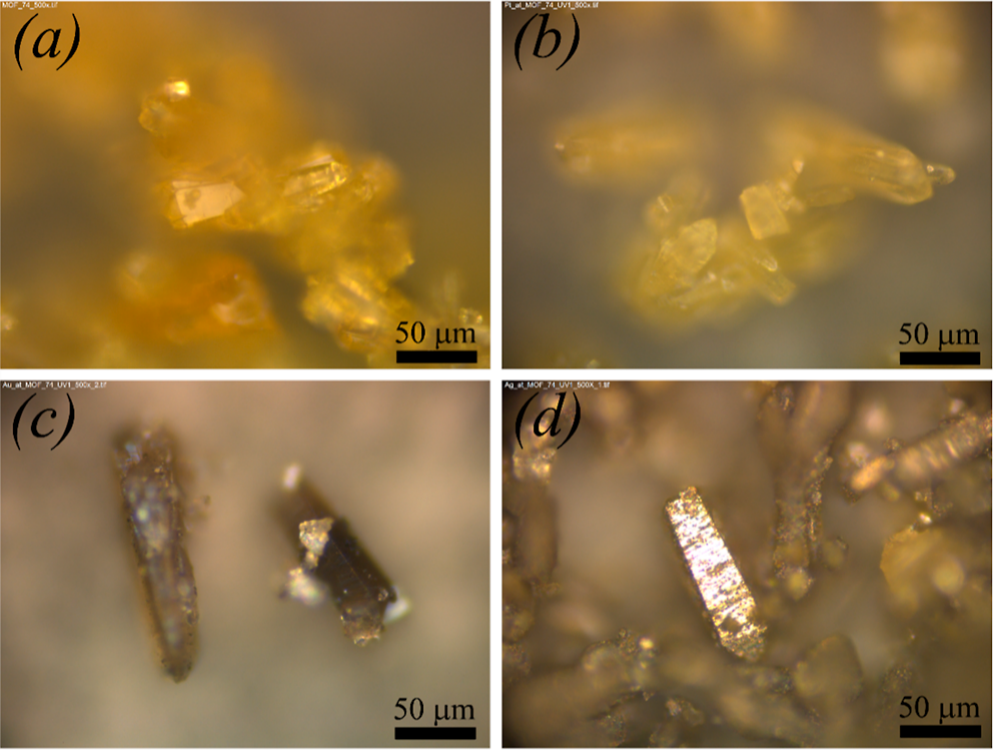
(a) Optical image of Zn-MOF-74 crystals obtained via solvothermal synthesis. Optical images of (b) PtNPs/Zn-MOF-74, (c) AuNPs/Zn-MOF-74, and (d) AgNPs/Zn-MOF-74 composites after the UV decoration process. Optical images were obtained using a LEICA DM 4500 P LED (80×) light microscope.

Zn-MOF-74 was synthesized using a previously reported direct solvothermal method with minor modifications.^12^ A 2,5-dihydroxyterephthalic acid solution (0.25 g, 26 mmol) in 25 mL of DMF was combined with a zinc nitrate hexahydrate solution (1.44 g, 4.84 mmol) in 25 mL of DMF. The mixture was stirred for 10 min. Then, 2.5 mL of deionized (DI) water was added to the mixture, and the obtained solution was transferred to a Teflon-lined autoclave, sealed, and heated at 100 °C for 20 h. After the solution was cooled, a powder of yellow hexagonal Zn-MOF-74 crystals was obtained (Figure 1a). This Zn-MOF-74 powder was initially suspended in DMF, and DMF was replaced with dimethyl sulfoxide 3 times; each replacement was done every 3 days. This method afforded 0.75 g of Zn-MOF-74 (82% yield).

#### 2.2.2 MeNPs/Zn-MOF-74 (Me = Pt, Au, and Ag) Composite Synthesis

MeNPs/Zn-MOF-74 (Me = Pt, Au, and Ag) composites were synthesized by reducing the metal precursor in the presence of a washed Zn-MOF-74 crystalline powder and an I-2959 photoinitiator under 350 nm (UVA) light. Upon UVA excitation, I-2959 affords ketyl radicals via the Norrish-type-I α-cleavage, which can reduce Pt^2+^, Au^3+^, and Ag^1+^ metal cations, forming NPs.^34^ In the synthesis pathway proposed herein, the metallic precursors and I-2959 photoinitiator were dissolved in DI water in the dark. Then, this solution and the Zn-MOF-74 crystalline powder (Figure 1a) were transferred to a UV reactor and irradiated with UVA light for 15 min. The final solution was removed from the UV reactor and filtered. The filtrate was sequentially washed with DI water and acetone for a few hours, filtered once more, and left to dry overnight in the dark. This synthesis method was performed using Pt^2+^, Au^3+^, and Ag^1+^ metal precursors with different concentrations and metal/Zn-MOF-74 mass ratios of ∼0.7 (low concentration synthesis, *UVI*) and ∼1.4 (high concentration synthesis, *UVII*). The synthesis for each MeNPs/Zn-MOF-74 (Me = Pt, Au, and Ag) composite is described in detail in the Supporting Information (SI). Figure 1b,c,d (and Figures S1, S2, and S3) show the optical images of PtNPs/Zn-MOF-74, AuNPs/Zn-MOF-74, and AgNPs/Zn-MOF-74 composites synthesized using the above-given method, respectively. Metallic NPs generated through the photoreduction process are distributed on the surface of Zn-MOF-74, as shown in the subsequent sections, in which PXRD, EDS, XPS, Raman, and high-resolution TEM (HRTEM) results are discussed. The change in the color of Zn-MOF-74 crystals observed in AuNPs/Zn-MOF-74 (Figure 1c) and AgNPs/Zn-MOF-74 (Figure 1d) composites is the first piece of evidence of the successful metal decoration/impregnation of Zn-MOF-74 crystals.

### 2.3 Instruments

PXRD data were collected using an Empyrean diffractometer equipped with a copper X-ray source (Cu Kα radiation, 45 kV, 40 mA) at within the 2θ range of 4–70° and variable integration time per step (1× to 4–13°, 5× to 13–33°, and 10× to 33–70°). Raman spectra were recorded using WITEC alpha 300 RA under 523 nm laser excitation (0.5 mW). SEM was performed using a Hitachi TM4000Plus microscope equipped with an Oxford energy-dispersive X-ray spectrometer. XPS was performed by using a monochromatic Al Kα (1486.6 eV) X-ray source in an ultrahigh vacuum system (Thermo Scientific ESCALAB QXi X-ray photoelectron spectrometer). HRTEM images were obtained using a Tecnai G2 FEI SuperTwin 200 kV. All experiments were performed at around 25 °C.

## 3 Results and Discussion

### 3.1 X-ray Diffraction

**Figure 2:**
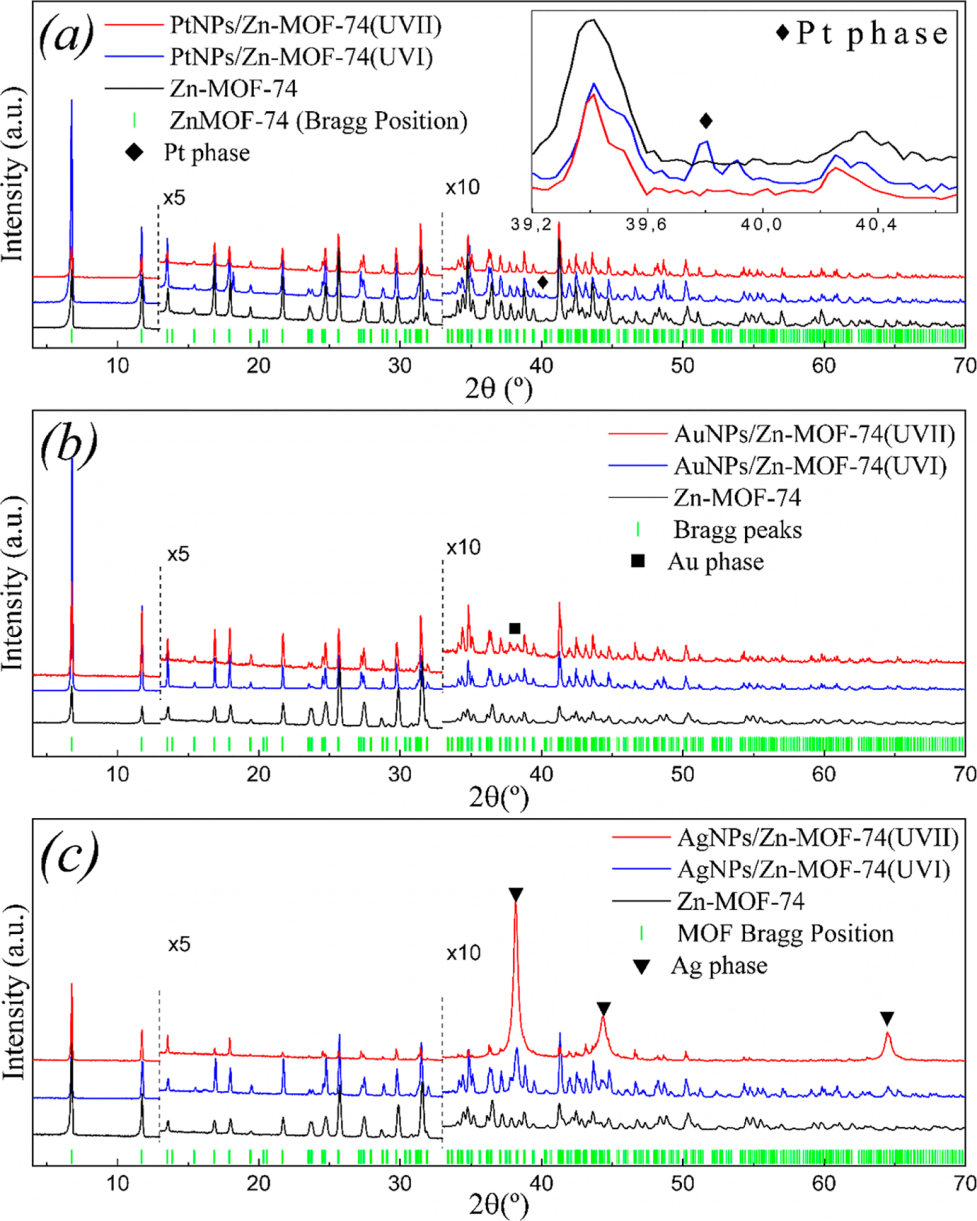
PXRD patterns of (a) Zn-MOF-74 (black), PtNPs/Zn-MOF-74 *UVI* (blue), and PtNPs/Zn-MOF-74 *UVII* (red) composites; (b) Zn-MOF-74 (black), AuNPs/Zn-MOF-74 *UVI* (blue), and AuNPs/Zn-MOF-74 *UVII* (red) composites; and (c) Zn-MOF-74 (black), AgNPs/Zn-MOF-74 *UVI* (blue), and AgNPs/Zn-MOF-74 *UVII* (red) composites. Integration times are between 4° < 2θ < 12° at 1× , 12° < 2θ < 32° at 5× , and 2θ > 32° at 10×. Bragg positions relative to the Zn-MOF-74 phase are shown in green bars. The peaks marked with ⧫, ■, and ▼ indicate the peaks of Pt (243678-ICSD), Au (64701-ICSD), and Ag (163723-ICSD) metal phases, respectively.

Figure 2 shows the PXRD patterns of pristine Zn-MOF-74 and PtNPs/Zn-MOF-74, AuNPs/Zn-MOF-74, and AgNPs/Zn-MOF-74 composites obtained via *UVI* and *UVII* syntheses and simulated patterns from the single-crystal data of Zn-MOF-74 and face-centered-cubic metallic Pt (243678-ICSD),^36^ Au (64701-ICSD),^37^ and Ag (163723-ICSD)^38^ phases. The analysis of these diffraction patterns shows that the crystallinity of Zn-MOF-74 is preserved in all MeNPs/Zn-MOF-74 (Me = Pt, Au, and Ag) composites after the metallic NP decoration/impregnation and washing processes. The characteristic peaks of metal phases can be observed in all but the XRD pattern of PtNPs/Zn-MOF-74 (*UVII*) composites.

Minor changes in the peak intensity and peak positions are observed in the PXRD patterns of *UVI* and *UVII* samples compared to the PXRD pattern of pristine Zn-MOF-74, as shown in Figures S4 and S5. Interestingly, the PXRD patterns of all MeNPs/Zn-MOF-74 (Me = Pt, Au, and Ag) *UVI* and *UVII* composites exhibit similar peak shifts and intensity changes. These results suggest that the changes in peak positions and intensities may be related to the washing process of the sample, i.e., to the exchange of molecules within Zn-MOF-74 pores.^39^

### 3.2 EDS and XPS

**Figure 3:**
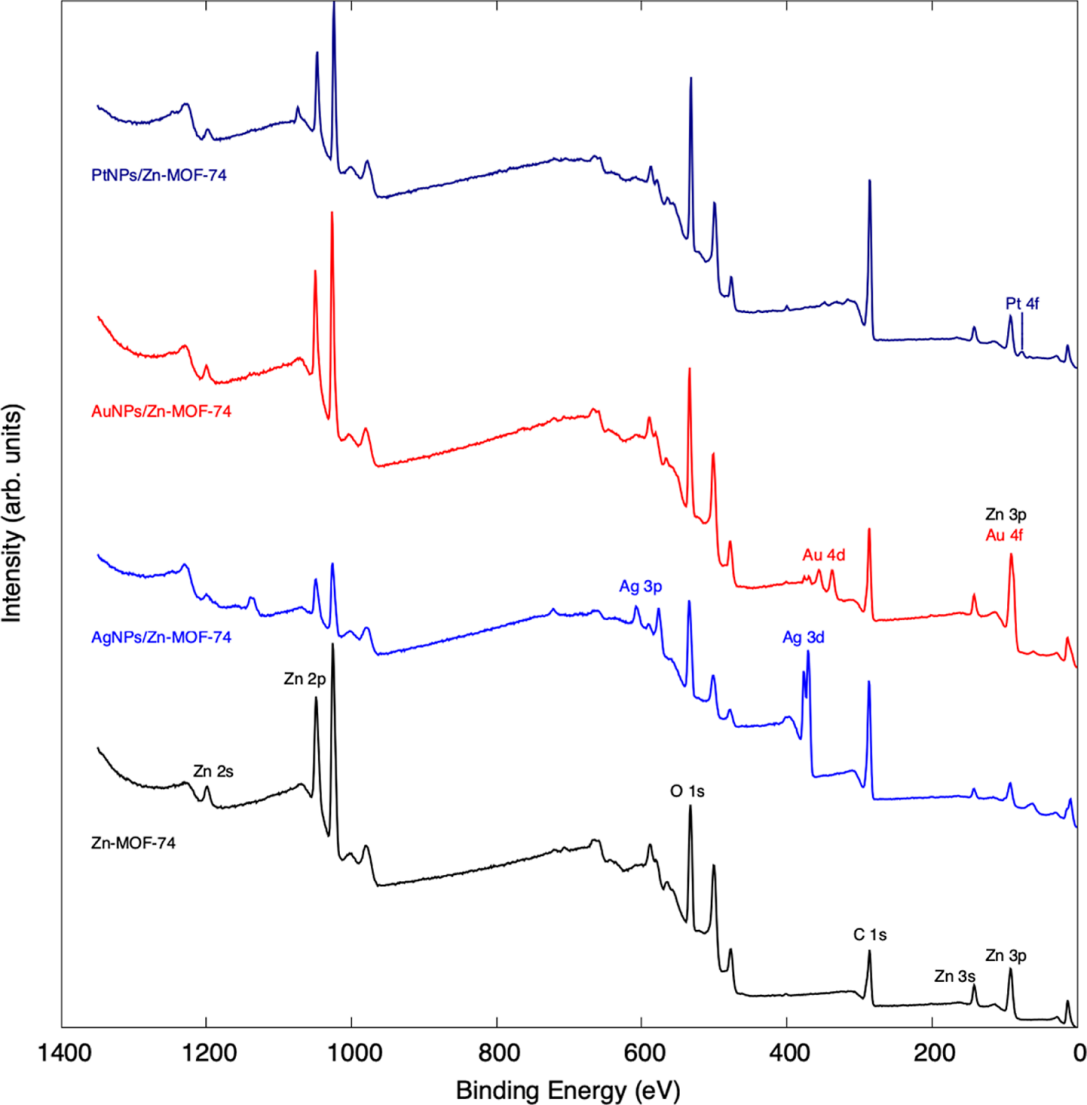
XPS spectra of PtNPs/Zn-MOF-74 (dark blue), AuNPs/Zn-MOF-74 (red), and AgNPs/Zn-MOF-74 composites and Zn-MOF-74 (black).

The presence of metallic NPs on MeNPs/Zn-MOF-74 (Me = Pt, Au, and Ag) composites was verified via EDS. The introduced metals in each decoration process were detected in all *UVI* and *UVII* samples, as shown in Figure S6. To further confirm the successful surface decoration of Zn-MOF-74 with Pt, Au, and Ag NPs, we performed XPS. Figure 3 shows the XPS survey spectra of pristine Zn-MOF-74 and MeNPs/Zn-MOF-74 (Me = Pt, Au, and Ag) composites. In the XPS spectrum of pristine Zn-MOF-74, the expected photoemission peaks of Zn, C, and O are observed. Remarkably, after the metal decoration process, the characteristic signatures of Zn-MOF-74 are still present in the XPS spectrum, indicating that the decoration process does not compromise the structural integrity of the MOF. In the survey XPS spectrum of PtNPs/Zn-MOF-74 composites, only the Pt 4f peak is observed. This is expected because the concentration of PtNPs within the XPS-probed area is notably low, only 0.2% of the respective metal content. In the survey XPS spectrum of AuNPs/Zn-MOF-74 composites, the presence of Au 4f and Au 3d peaks confirms the successful deposition of AuNPs on Zn-MOF-74, with an NP concentration of ∼4%. In the survey XPS spectrum of AgNPs/Zn-MOF-74 composites, the Ag 3d and Ag 3p peaks are distinctly visible, proving the decoration of Zn-MOF-74 with AgNPs. As estimated from the survey XPS spectrum, the concentration of AgNPs in the XPS-probed area is ∼5%.

### 3.3 Raman Spectroscopy

**Figure 4:**
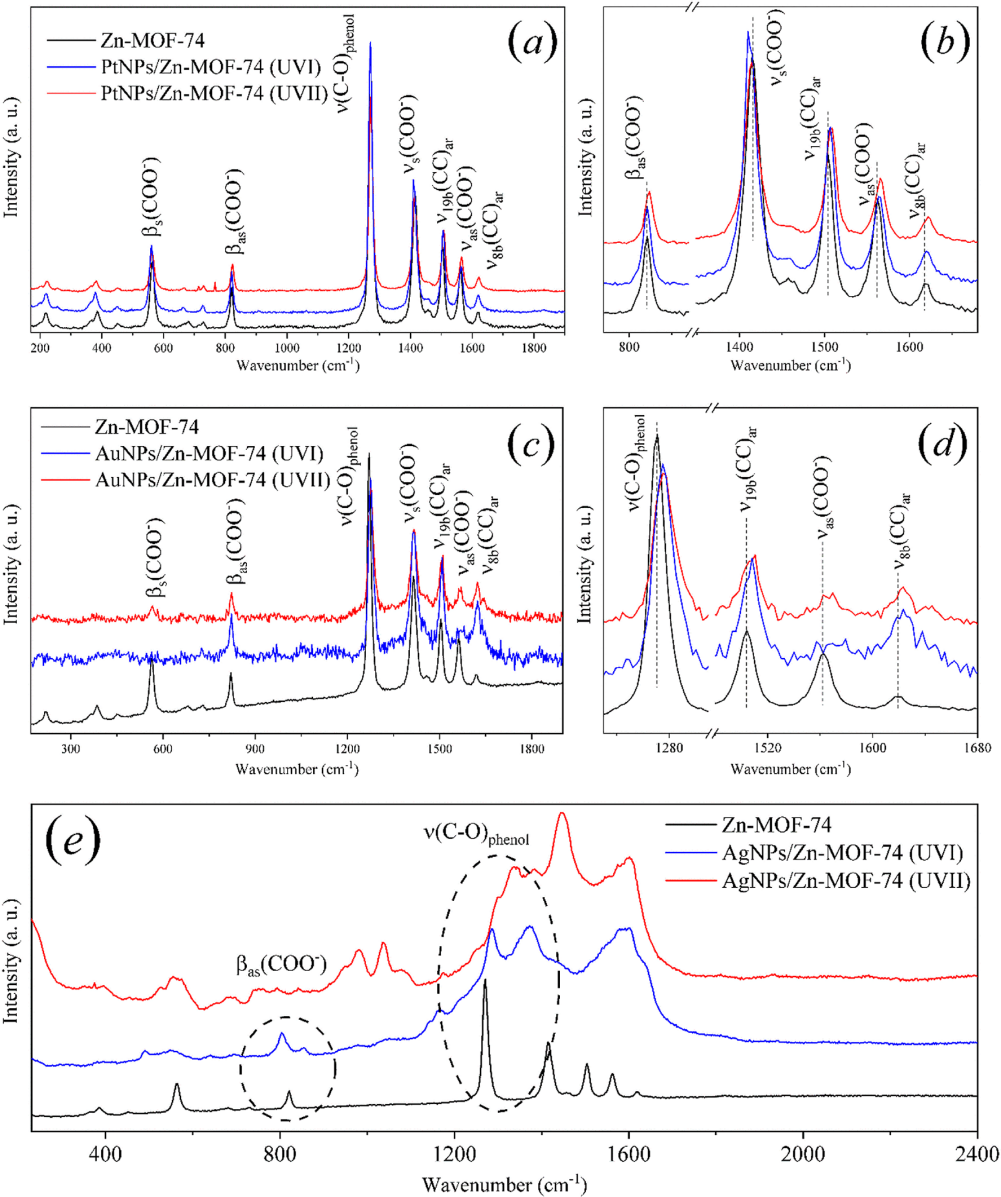
Raman spectra of Zn-MOF-74 (black) and MeNPs/Zn-MOF-74 *UVI* and *UVII* composites (blue and red, respectively). (a) PtNPs/Zn-MOF-74 composites in the relevant wavelength spectra range, and (b) peak shifts of PtNPs/Zn-MOF-74 composites. (c) AuNPs/Zn-MOF-74 composites in the relevant wavelength spectra range and (d) peak shifts of AuNPs/Zn-MOF-74 composites. (e) AgNPs/Zn-MOF-74 composites in the relevant wavelength spectra range.

Figure 4 displays the Raman spectra of pristine Zn-MOF-74 and MeNPs/Zn-MOF-74 (Me **=** Pt, Au, and Ag) composites obtained via *UVI* and *UVII* syntheses. In the Raman spectrum of Zn-MOF-74, the observed vibration modes are characteristic of the Zn-MOF-74 structure, corresponding to the ν(COO^–^), ν(C–O)_phenol_, and ν(CC)_ar_ modes of carboxylate, phenolate, and aromatic ring groups, respectively.^40^ In the Zn-MOF-74 structure, the oxygen atoms in the phenolate and carboxylate groups coordinate with metallic cations to form the SBU, making them sensitive to the structural modification of Zn-MOF-74.^41^

A comparison of the Raman spectra of Zn-MOF-74 and PtNPs/Zn-MOF-74 (Figure 4a) and the AuNPs/Zn-MOF-74 composites (Figure 4c) shows no significant changes in the mode frequencies or intensities. The minor frequency shifts (Table S1) observed for the signals of ν_s_(COO^–^), ν_19b_(CC)_ar_, ν_as_(COO^–^), and ν_8b_(CC)_ar_ modes, as shown in the insets of Figure 4b,d, may be attributed to the surface order rearrangement during the composite production or solvent changes within Zn-MOF-74 cavities due to the washing process. Importantly, no changes are observed in the 3D structure of Zn-MOF-74 crystals, as evidenced by the PRXD pattern of the MeNPs/Zn-MOF-74 samples shown in Figure 2. Additionally, no significant differences are observed in the Raman spectra of PtNPs/Zn-MOF-74 composites produced via *UVI* and *UVII* processes. Consequently, there is no correlation between the shifts in the vibrational mode frequencies and platinum concentration during the synthesis.

The Raman spectra of AuNPs/Zn-MOF-74 composites (Figure 4c) show the same characteristics as those of PtNPs/Zn-MOF-74 composites, with minor peak frequency shifts in some observed modes. However, the reduction in the intensity of β_s_(COO^–^) and ν_as_(COO^–^) mode signals in the Raman spectra of AuNPs/Zn-MOF-74 *UVI* and *UVII* composites indicates that the Au layer on the Zn-MOF-74 crystal surface (Figure 1c) has affected the Zn-MOF-74 structure in the area probed by laser during Raman experiments (Figure 4d).

Figure 4e shows the Raman spectra of the AgNPs/Zn-MOF-74 composites. This case differs from the preview spectra (Figure 4a–d) because the Raman spectra of AgNPs/Zn-MOF-74 composites are considerably affected by the Ag decoration/incorporation processes (Figure 1d), which increase the intensity of ν_s_(COO^–^), ν_19b_(CC)_ar_, ν_as_(COO^–^), and ν_8b_(CC)_ar_ signals. This result indicates a remarkable rearrangement of the Zn-MOF-74 structure in the volume probed by the laser during Raman experiments. Notably, the crystal structure of Zn-MOF-74 is not affected by the Ag decoration-incorporation process, as demonstrated by the XRD pattern of AgNPs/Zn-MOF-74 composites (Figure 2c).

### 3.4 SEM and TEM

**Figure 5:**
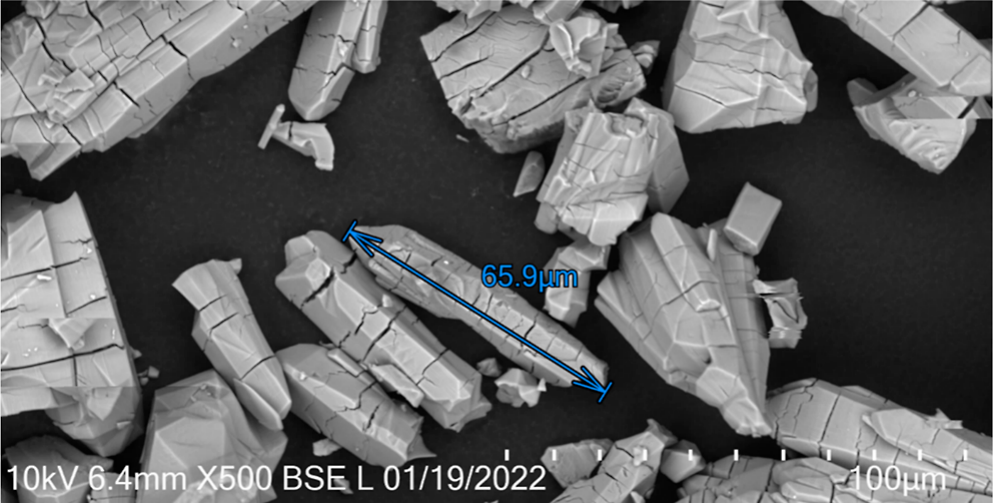
BSE–SEM image of Zn-MOF-74 obtained via the solvothermal method.

The morphological analysis of Zn-MOF-74 was performed by using SEM with backscattered electron (BSE) detection. As shown in the BSE–SEM image in Figure 5, Zn-MOF-74 obtained via the solvothermal method is microsized and has a rod-like morphology.^9,14,15^ The cracks in the Zn-MOF-74 crystal observed in Figure 5 are caused by the electron beam interaction during imaging acquisition.

**Figure 6:**
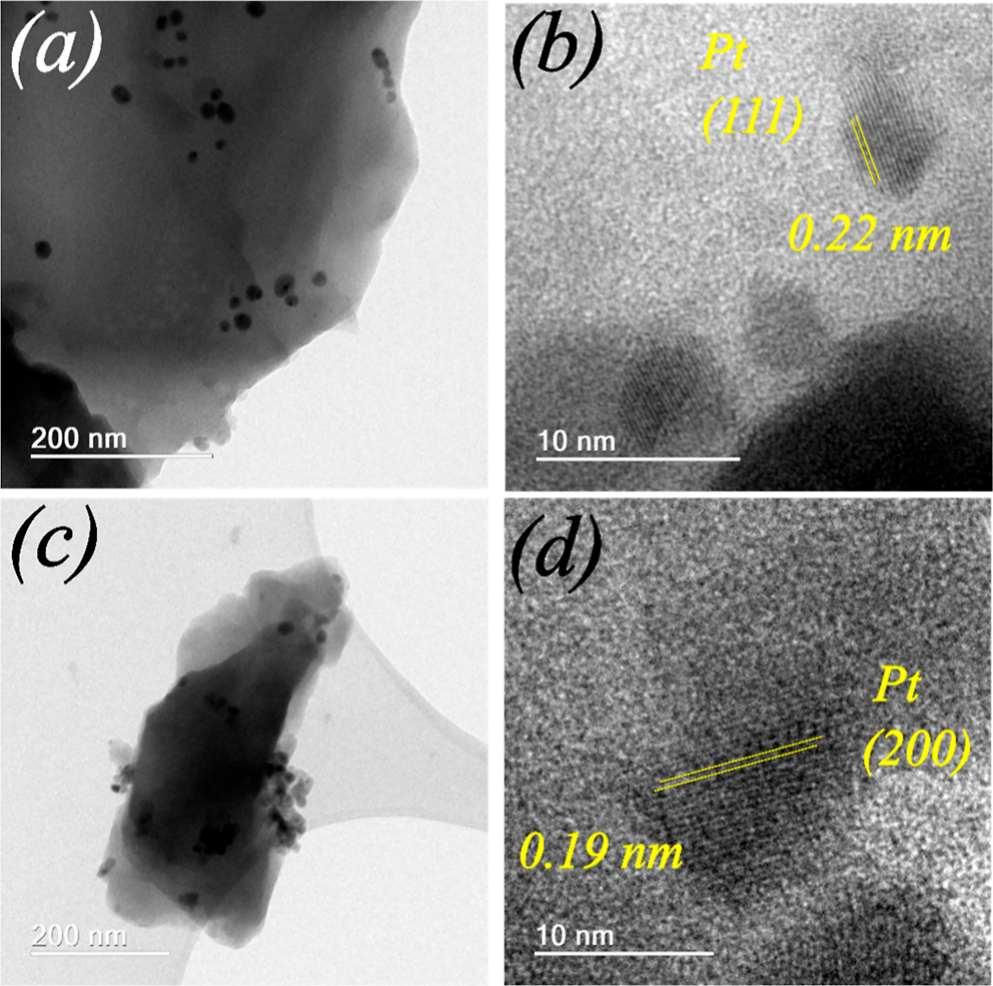
HRTEM images of PtNPs/Zn-MOF-74 (*UVI*) (a,b) and PtNPs/Zn-MOF-74 (*UVII*) (c,d) composites. (d) PtNPs were identified by comparing the interference fringes and Bragg plane distances of Pt crystals (ICSD 243678).

**Figure 7:**
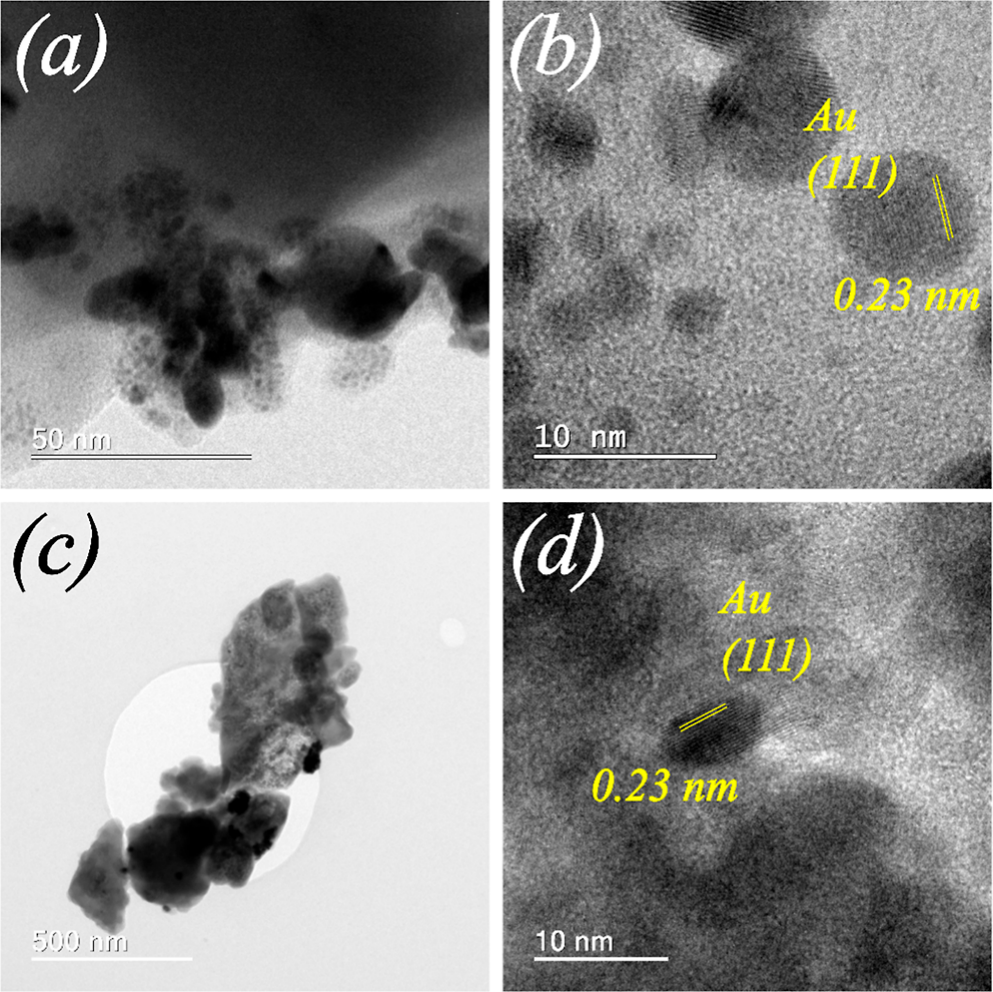
HRTEM images of AuNPs/Zn-MOF-74 (*UVI*) (a,b) and AuNPs/Zn-MOF-74 (*UVII*) (c,d) composites. (d) AuNPs were identified by comparing the interference fringes and Bragg plane distances of the Au crystal (ICSD 64701).

**Figure 8:**
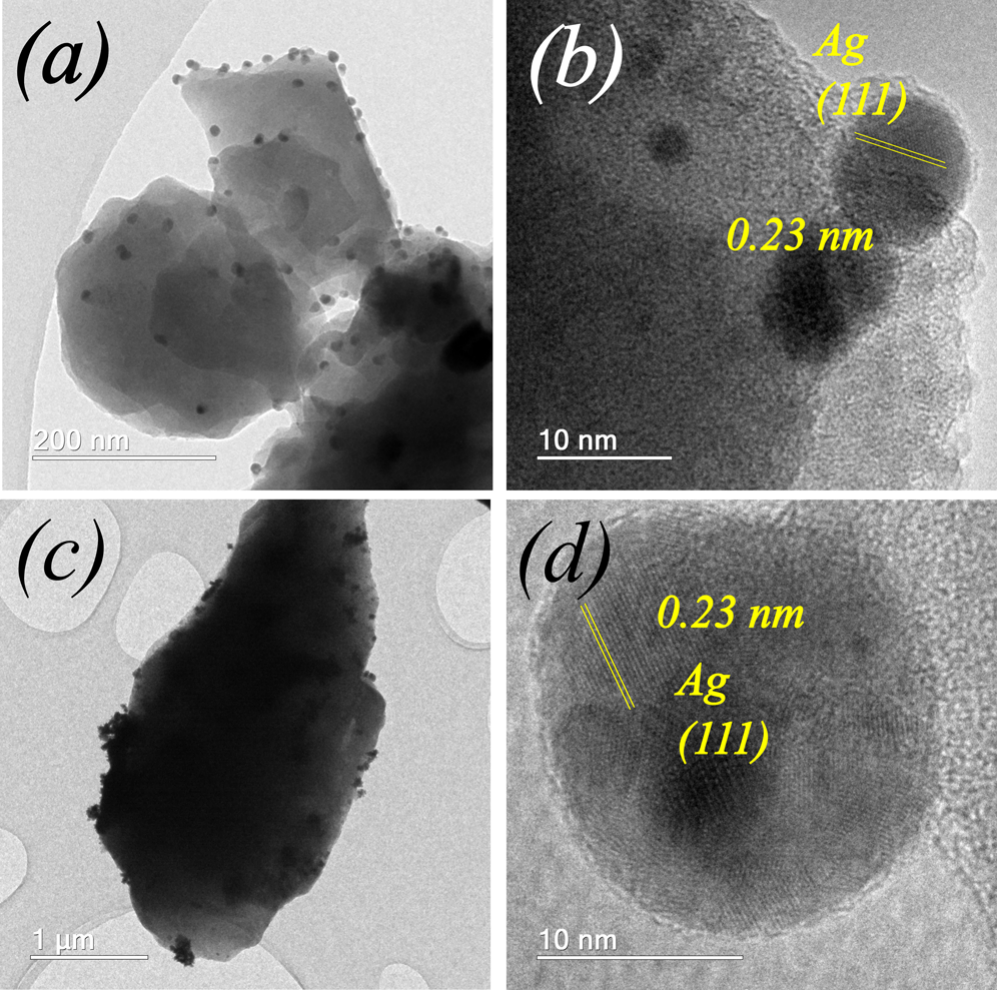
HRTEM images of AgNPs/Zn-MOF-74(*UVI*) (a,b) and AgNPs/Zn-MOF-74(*UVII*) (c,d) composites. (d) AgNPs were identified by comparing the interference fringes and Bragg plane distances of Ag crystals (ICSD 163723).

In the HRTEM images in Figures 6–8, metallic NPs are visible in all MeNPs/Zn-MOF-74 (Me = Pt, Au, and Ag) composites produced using the I-2959 photoinitiator under UV irradiation. Additional chemical characterizations of NPs were performed by using EDS (Figure S6). In low (*UVI*) and high (*UVII*) metal concentration synthesis, NPs decorating Zn-MOF-74 have a spherical shape and are randomly distributed on the Zn-MOF-74 surface (Figures 6b, 7b, and 8b). According to the statistical analysis of HRTEM images performed using a log–normal function (Figure S7), Pt and Au NPs decorating Zn-MOF-74 obtained via the *UVI* synthesis procedure (metal/Zn-MOF-74 mass ratio = ∼ 0.7) have diameters of approximately 2.41 ± 0.75 and 2.21 ± 1.31 nm, respectively. However, Pt and Au NPs decorating Zn-MOF-74 obtained via the *UVII* synthesis procedure (metal/Zn-MOF-74 mass ratio = ∼1.4) have diameters of approximately 9.89 ± 4.74 and 4.86 ± 2.51 nm, respectively. These results demonstrate that during syntheses using the I-2959 photoinitiator and UV irradiation procedure, higher metal concentrations afford larger metallic NPs. Similar findings have been reported on the influence of the metal concentration on an increase in the diameter of AuNPs synthesized via photochemical metal deposition on oxidized single-walled carbon nanotubes.^42^ These results show that high metal concentrations promote aggregation and the formation of larger metallic NPs upon UV photoreduction without needing a surfactant to control the particle size.^43^ AgNPs/Zn-MOF-74 composites obtained at low metal precursor concentrations (*UVI*) show NPs with a bimodal size distribution with average sizes of 2.39 ± 0.31 and 10.82 ± 0.27 nm. However, AgNPs/Zn-MOF-74 composites obtained with high metal precursor concentrations (*UVII*) show NPs with an average size of 4.36 ± 2.32 nm.

Figures 6–8 show the HRTEM interference fringes of metallic NPs on the Zn-MOF-74 surface produced using the I-2959 photoinitiator and UV irradiation. The distances between the interference fringes of PtNPs (Figure 6b,d) are 0.22 and 0.19 nm, respectively. These values correspond to the (111) and (200) plane distances within face-centered cubic Pt crystal, respectively^36^ [ICSD 243678]. In the case of Au and Ag NPs, the distances between the interference fringes (Figures 7d, 8d) are 0.23 nm, which is characteristic of the (111) plane distance within face-centered cubic Au (ICSD 64701)^37^ and Ag (ICSD 163723)^38^ crystals. Figures S8, S9 and S10 show the HRTEM images and the corresponding FFT patterns of selected nanoparticles evidenceing the (111) and (200) planes symmetry.

According to the XRD patterns shown in Figure 2, the synthesis of MeNPs/Zn-MOF-74 (Me = Pt, Au, and Ag) composites, performed in the presence of the washed Zn-MOF-74 powder and I-2959 photoinitiator with different concentrations of Pt, Au, and Ag metallic precursors under UV irradiation, does not affect the overall 3D structure of Zn-MOF-74. After decoration, Zn-MOF-74 crystals remain undamaged, and no metallic NPs or metallic ions are found within Zn-MOF-74 cavities. Notably, PXRD patterns do not show peaks associated with PtNPs obtained during the *UVII* synthesis (Figure 2a). However, XPS (Figure 3), EDS (Figure S6e), and HRTEM (Figure 6) results show the composition and shape of NPs in PtNPs/Zn-MOF-74 composites.

Raman experiments reveal that the crystal surfaces reorganize upon metal decoration. The surface reorganization is more pronounced in AgNPs/Zn-MOF-74 composites compared with AuNPs/Zn-MOF-74 and PtNPs/Zn-MOF-74 composites. This difference is likely due to the smaller standard reduction potential of Ag^+^ (0.8 V) compared to those of Pt^2+^ (1.2 V) and Au^3+^ (1.7 V), resulting in a higher amount of Ag(s) deposited on the surface of composites, as evidenced by the color change from yellowish (pristine Zn-MOF-74) to metallic (AgNPs/Zn-MOF-74), as shown in Figure 1d. An analysis of the HRTEM images (Figures 6–8) of the different samples reveals that NPs produced in the photoreduction process are spherical, as observed in other similar reports for this type of reduction.^34^

## 4 Conclusions

This study introduces a novel and robust method for decorating Zn-MOF-74 crystals with Pt, Au, and Ag NPs. This is achieved via the photoreduction of metallic precursors in a solution containing Zn-MOF-74 crystals, which are exposed to UV light in the presence of the Irgacure photoinitiator. X-ray and Raman analyses confirm the ability of the Zn-MOF-74 crystals to endure the growth and decoration of Pt, Au, and Ag NPs. XPS and EDS provide additional evidence of the chemical composition of MeNPs/Zn-MOF-74 (Me **=** Pt, Au, and Ag) composites.

The HRTEM images of MeNPs/Zn-MOF-74(Me **=** Pt, Au, and Ag) composites show the presence of spherical crystalline Pt, Au, and Ag NPs over the Zn-MOF-74 crystal surface. The size distribution analysis of NPs indicates that MeNPs/Zn-MOF-74 (Me **=** Pt, Au, and Ag) composites obtained during the synthesis with higher metal concentrations exhibit larger metallic NPs and more agglomerates over the Zn-MOF-74 surface than those with low metal concentrations under the same UV irradiation conditions. Notably, while NPs with a size of ∼1 nm are identified over the Zn-MOF-74 surface, no metallic NPs are observed within the pores of the Zn-MOF-74 crystals.

## Supporting Information Available

Detailed description of the MeNPs/Zn-MOF-74 (Me = Pt, Au, and Ag) synthesis with low (*UVI*) and high (*UVII*) metal concentrations and additional PXRD, EDS, XP, Raman, and HRTEM data (PDF)

## Footnotes

1 2-Hydroxy-4′-(2-hydroxyethoxy)-2-methylpropiophenone.
