# Supplementary material for: Morphological and Structural Characterization of (Pt, Au, and Ag) Nanoparticle/Zn-MOF-74 Composites
Source: ACS Omega. 2024 May 10;9(20):21939–47. doi: 10.1021/acsomega.3c09973 (PMC11112587; doi:10.1021/acsomega.3c09973)
Supplement: Supplementary file 1 — ao3c09973_si_001.pdf [file ao3c09973_si_001.pdf]

Morphological and structural characterization of (Pt, Au, Ag) NPs/Zn-MOF-74 composites

Juliana Assunção Pereira de Figueiredo[1]*, Maximiliano Jesús Moreno Zapata*[1]*, Laíse Serra Amorim*[1]*, João Alves de Oliveira Neto*[1]*, Douglas Rodrigues Miquita*[2]*, Edmar Avellar Soares*[1]*, Karla Balzuweit*[1]*, and Carlos Basílio Pinheiro*[1]*

[1]Physics Department, Universidade Federal de Minas Gerais, Belo Horizonte, 31270-901, Brazil. [2]Centro de Microscopia, Universidade Federal de Minas Gerais, Belo Horizonte, 31270-901, Brazil

TABLE OF CONTENTS

1. SYNTHESIS 3
2. POWDER X-RAY DIFFRACTION 5
3. EDS 8
4. XPS 12
5. RAMAN 13
6. HRTEM 13

LIST OF FIGURES

Figure S1: Optical image of the (a) PtNPs/Zn-MOF-74(UVI) and (b) PtNPs/Zn-MOF-74(UVII). Images were obtained using a Light Microscope LEICA DM 4500 P LED. 3
Figure S2: Optical image of the (a) AuNPs/Zn-MOF-74(UVI) and (b)AuNPs/Zn-MOF-74(UVII). Images were obtained using a Light Microscope LEICA DM 4500 P LED. 4
Figure S3: Optical image of the (a) AgNPs/Zn-MOF-74(UVI) and (b)AgNPs/Zn-MOF-74(UVII). Images were obtained using a Light Microscope LEICA DM 4500 P LED. 5
Figure S4: Comparison of the experimental PXRD pattern of the composites: PtNPs/Zn-MOF-74 (UVI) (purple), PtNPs/Zn-MOF-74 (UVII) (dark purple), AuNPs/Zn-MOF-74 (UVI) (red), PtNPs/Zn-MOF-74 (UVII) (dark red), AgNPs /Zn-MOF-74 (UVI) (dark blue), AgNPs /Zn-MOF-74 (UVII) (light blue) 6
Figure S5: XRD of the composite MeNPs/Zn-MOF-74 (Me = Ag, Au, Pt) produced using different metal precursors concentrations and of pure Zn-MOF-74. No remarkable displacement in the peak's positions is observed. 7
Figure S6: EDS spectra of the MeNPs/Zn-MOF-74 (Me = Ag, Au, Pt) composites in all cases. It was possible to identify the metal presence: (a) AuNPs/Zn-MOF-74 (UVI), (b)AuNPs/Zn-MOF-74 (UVII), (c) AgNPs/Zn-MOF-74 (UVI), (d) AgNPs/Zn-MOF-74 (UVII) and (e) PtNPs/Zn-MOF-74 (UVII) 12
Figure S7: Size distribution of the particles in the (a) PtNPs/Zn-MOF-74 (UVI), (b) PtNPs/Zn-MOF-74 (UVII), (c) AuNPs/Zn-MOF-74 (UVI), (d) AuNPs/Zn-MOF-74 (UVII), (e) AgNPs/Zn-MOF-74 (UVI) and (f) AgNPs/Zn-MOF-74 (UVII) 15
Figure S8: HRTEM image and the FFT pattern of the selected Pt nanoparticle image 16
Figure S9: HRTEM image and the FFT pattern of the selected Au nanoparticle image. 16
Figure S10: HRTEM image and the FFT pattern s of the selected Ag nanoparticle image.... 17

LIST OF TABLES

Table S1: Assignment of the Raman spectra vibrational modes for Zn-MOF-74 and PtNPs/Zn-MOF-74 composite. 13
Table S2: Assignment of vibrational bands from Raman spectra of Zn-MOF-74 and AuNPs/Zn-MOF-74 composite. 13

1. SYNTHESIS

The PtNPs/Zn-MOF-74 synthesis was performed in the presence of washed Zn-MOF-74 powder and I-2959 photoinitiator at different concentrations of the $Pt(acac)_2$. **Synthesis *UVI*:** $Pt(acac)_2$ (0.013 g, 0.033 mmol) was solubilized in 10 mL of deionized (DI) water, and I-2959 (0.008 g, 0.036 mmol) was dissolved in 10 mL DI water in the dark. 10 mg of the Zn-MOF-74 powder and the $Pt(acac)_2$ solution was transferred to the UV reactor, and then the I-2959 solution was added. The final solution was stirred for 15 minutes under UV irradiation, removed from the UV reactor, and filtered. The filtrated was washed with DI water and then with acetone for a few hours, filtered once more, and left to dry overnight, in the dark. 4 mg of PtNPs/Zn-MOF-74 nanocomposite (yield 40%) was obtained (Figure 1a). **Synthesis *UVII*:** $Pt(acac)_2$ (0.027 g, 0.069 mmol) was solubilized in 700 µL of DI water, and I-2959 (0.017 g, 0.076 mmol) was dissolved in 2.5 mL of DI water in the dark. 40 mg of the Zn-MOF-74 powder and the $Pt(acac)_2$ solution was transferred to the UV reactor, and then the I-2959 solution was added. The final solution was stirred for 15 minutes under UV irradiation, removed from the UV reactor, and filtered. The filtrated was washed with DI water and then with acetone for a few hours, filtered once more, and then left to dry overnight, in the dark. 19 mg PtNPs/Zn-MOF-74 nanocomposite (yield 48 %) was obtained (Figure 1b).

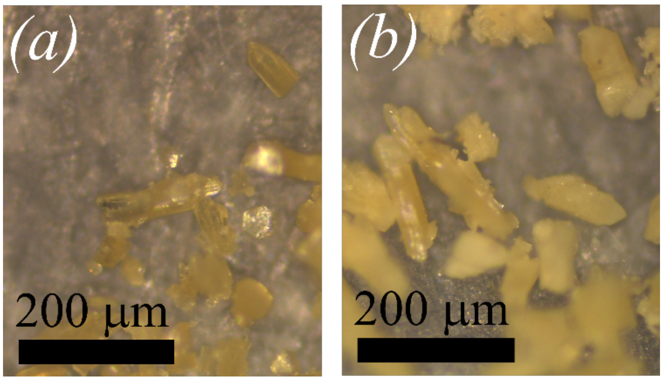

Figure S1: Optical image of the (a) PtNPs/Zn-MOF-74(UVI) and (b) PtNPs/Zn-MOF-74(UVII). Images were obtained using a Light Microscope LEICA DM 4500 P LED.

The AuNPs/Zn-MOF-74 synthesis was performed in the presence of washed Zn-MOF-74 powder and I-2959 photoinitiator at different concentrations of the metallic precursor $HAuCl_4$. **Synthesis *UVI*:** $NaOH_{(w)}$ (0.1 M) was dropped in 330 µL of the $HAuCl_4$ (0.1 M) solution (0.033 mmol) until pH = 12. I-2959 (0.008 g, 0.036 mmol) was dissolved in 10 mL of DI water in the dark. 10 mg of the Zn-MOF-74 powder and the metallic precursor solution were transferred to the UV reactor, and then the I-2959 solution was added. The final solution was stirred for 15 minutes under UV irradiation, removed from the UV reactor, and filtered. The filtrated was washed with DI water and then with acetone for a few hours, filtered once more, and left to dry overnight, in the dark. 5 mg AuNPs/Zn-MOF-74 nanocomposite (yield 50%) was obtained (Figure 2a). **Synthesis *UVII*:** $NaOH_{(w)}$ (0.1 M) was dropped in 700 µL of the $HAuCl_4$ (0.1 M) solution (0.033 mmol) until pH=12. I-2959 (0.017 g, 0.076 mmol) was dissolved in 2.5 mL of DI water in the dark. 40 mg of the Zn-MOF-74 powder and the metallic precursor solution were transferred to the UV reactor, and then the I-2959 solution was added. The final solution was stirred for 15 minutes under UV irradiationn, removed from the UV reactor, and filtered. The filtrated was washed with DI water and then with acetone for a few hours, filtered once more, and left to dry overnight, in the dark. 27 mg AuNPs/Zn-MOF-74 nanocomposite (yield 68%) was obtained (Figure 2b).

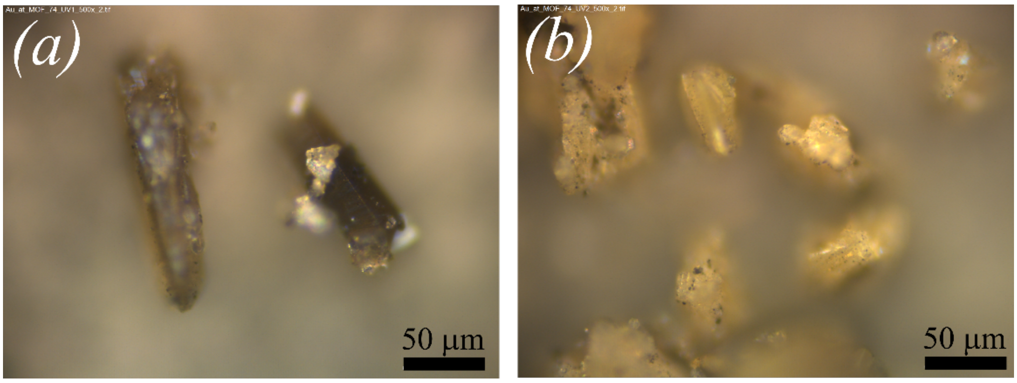

Figure S2: Optical image of the (a) AuNPs/Zn-MOF-74(UVI) and (b)AuNPs/Zn-MOF-74(UVII). Images were obtained using a Light Microscope LEICA DM 4500 P LED.

The AgNPs/Zn-MOF-74 synthesis was performed in the presence of washed Zn-MOF-74 powder and I-2959 photoinitiator at different concentrations of the metallic precursor $AgNO_3$. **Synthesis *UVI*:** $AgNO_3$ (0.0056 g, 0,033 mmol) was solubilized in 10 mL of DI water, and I-2959 (0.008 g, 0.036 mmol) was dissolved in 10 mL of DI water in the dark. 10 mg of the Zn-MOF-74 powder and the $AgNO_3$ solution were transferred to the UV reactor, and then the I-2959 solution was added. The final solution was stirred for 15 minutes under UV irradiation, removed from the UV reactor, and filtered. The filtrated was washed with DI water and then with acetone for a few hours, filtered once more, and left to dry overnight, in the dark. 5 mg AgNPs/Zn-MOF-74 nanocomposite (yield 50 %) was obtained (Figure 3a). **Synthesis *UVII*:** $AgNO_3$ (0.012 g, 0.069 mmol) was solubilized in 700 μL of DI water, and I-2959 (0.017 g, 0.076 mmol) was dissolved in 2.5 mL of DI water in the dark. 40 mg of the Zn-MOF-74 powder and the $AgNO_3$ solution were transferred to the UV reactor, and then the I-2959 solution was added. The final solution was stirred for 15 minutes under UV irradiation, removed from the UV reactor, and filtered. The filtrated was washed with DI water and then with acetone for a few hours, filtered once more, and left to dry overnight, in the dark. 28 mg AgNPs/Zn-MOF-74 nanocomposite (yield 70 %) was obtained (Figure 3b).

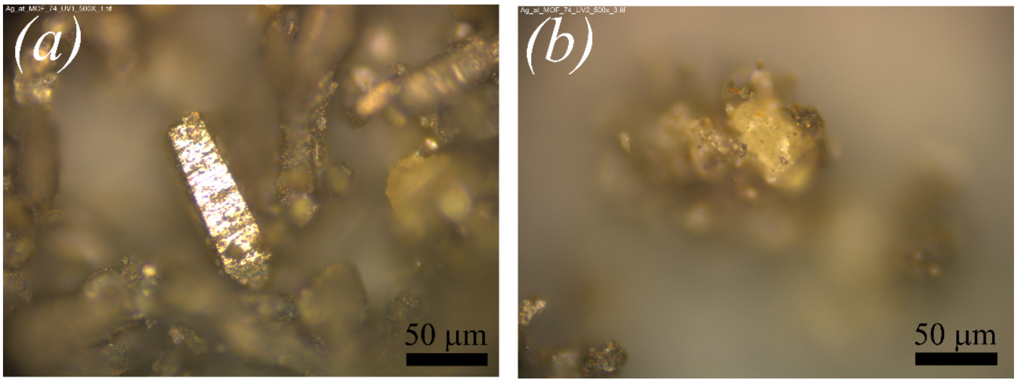

Figure S3: Optical image of the (a) AgNPs/Zn-MOF-74(UVI) and (b)AgNPs/Zn-MOF-74(UVII). Images were obtained using a Light Microscope LEICA DM 4500 P LED.

2. POWDER X-RAY DIFFRACTION

Figures S4 and S5 compare the powder X-ray diffraction pattern of Zn-MOF-74 and MeNPs/Zn-MOF-74 (Me = Ag, Au, Pt) composites. In all samples, the crystalline characteristic was preserved. The cell parameters of Zn-MOF-74 calculated using Bragg's law in a hexagonal system are $a = b = 26.123$ (25) Å and $c = 6.773$ (3) Å. The cell parameters of the MeNPs/Zn-MOF-74 (Me = Pt, Au, Ag) composites did not show any relevant deviations from the previous values.

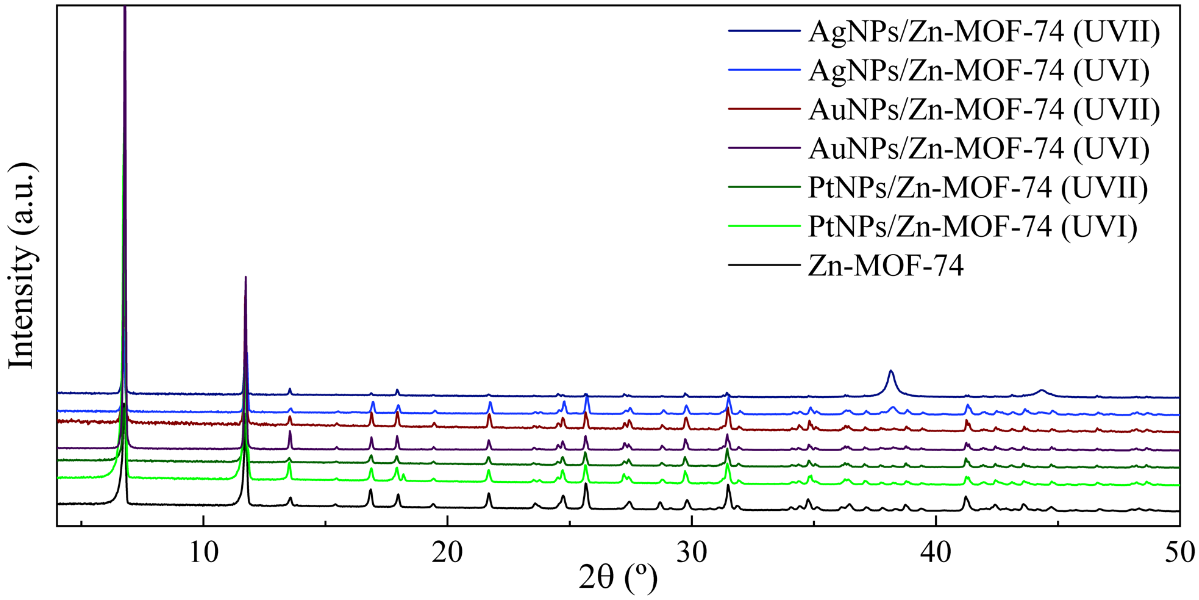

Figure S4: Comparison of the experimental PXRD pattern of the composites: PtNPs/Zn-MOF-74 (UVI) (purple), PtNPs/Zn-MOF-74 (UVII) (dark purple), AuNPs/Zn-MOF-74 (UVI) (red), PtNPs/Zn-MOF-74 (UVII) (dark red), AgNPs /Zn-MOF-74 (UVI) (dark blue), AgNPs /Zn-MOF-74 (UVII) (light blue).

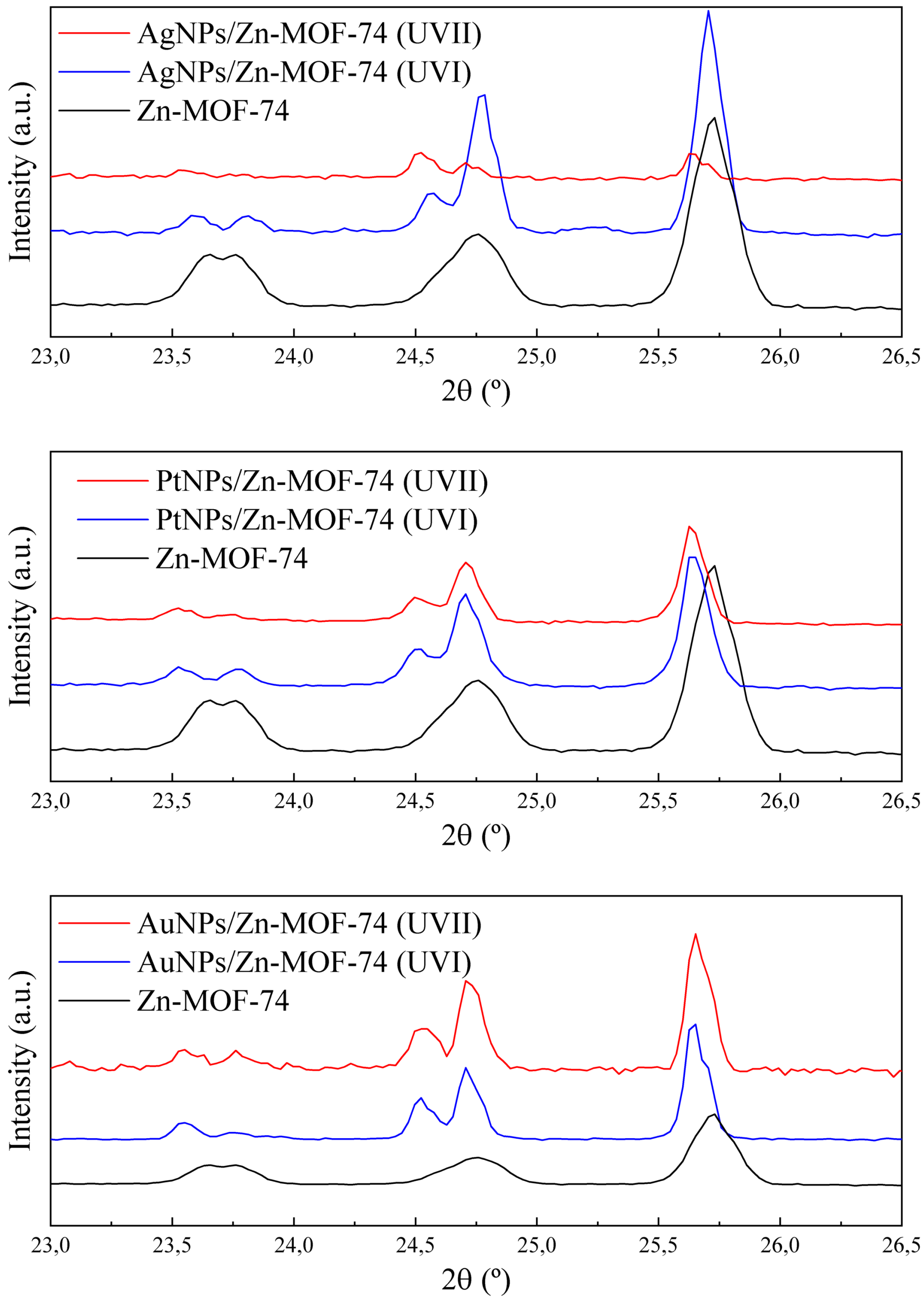

Figure S5: XRD of the composite MeNPs/Zn-MOF-74 (Me = Ag, Au, Pt) produced using different metal precursors concentrations and of pure Zn-MOF-74. No remarkable displacement in the peak's positions is observed.

3. EDS

Figures S6a-e show the EDS spectra of MeNPs/MOF-74 (Me = Pt, Au, Ag), evidencing the presence of the metals in their respective composites.

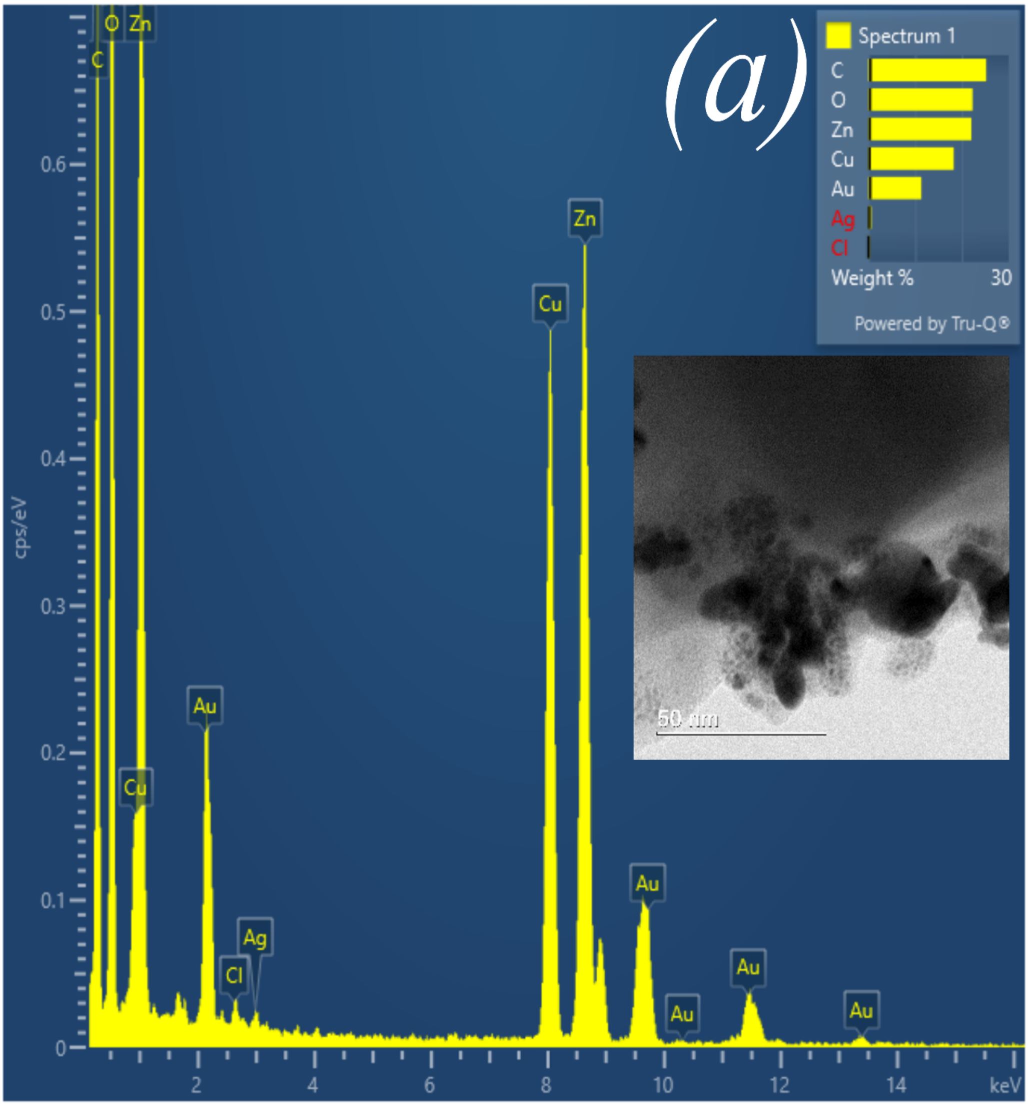

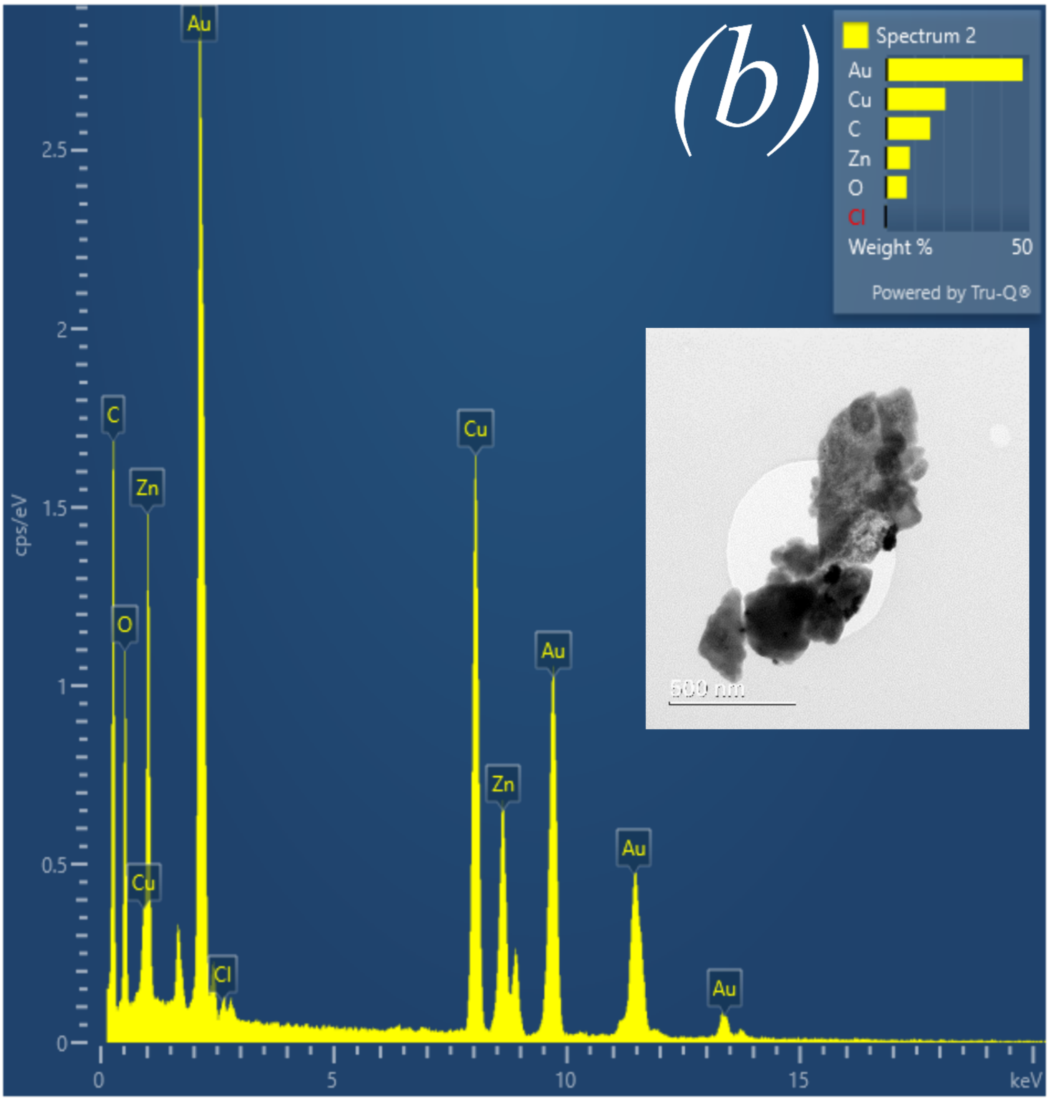
(b)
Spectrum 2
Au
Cu
C
Zn
O
Cl
Weight %
50
Powered by Tru-Q®
500 nm
cps/eV
keV
Au
C
Zn
O
Cu
Cl
Cu
Zn
Au
Au
Au

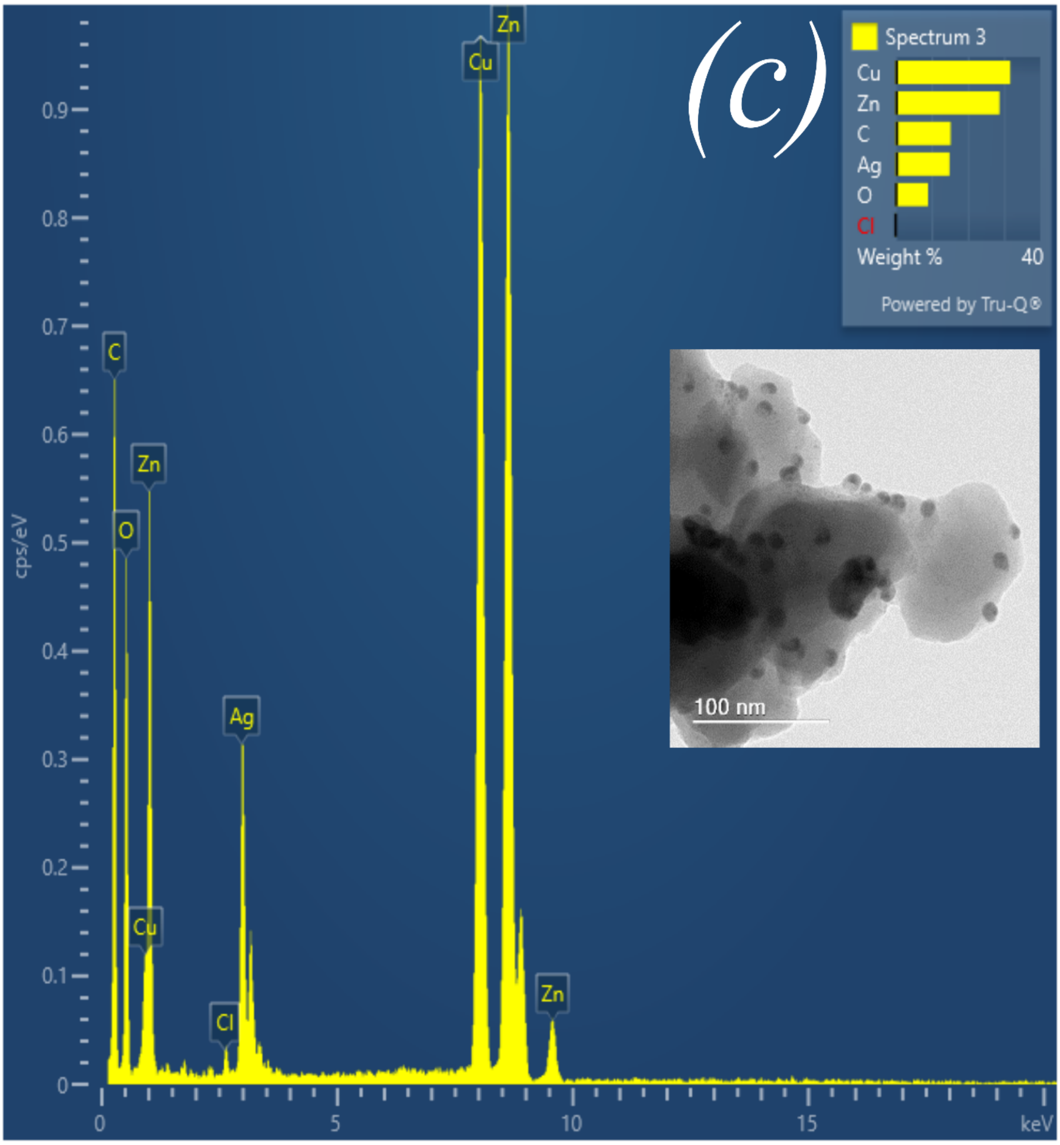
(c)
Spectrum 3
Cu
Zn
C
Ag
O
Cl
Weight %
40
Powered by Tru-Q®
100 nm
cps/eV
0.9
0.8
0.7
0.6
0.5
0.4
0.3
0.2
0.1
0
0
5
10
15
keV
C
Zn
O
Cu
Cl
Ag
Cu
Zn
Zn

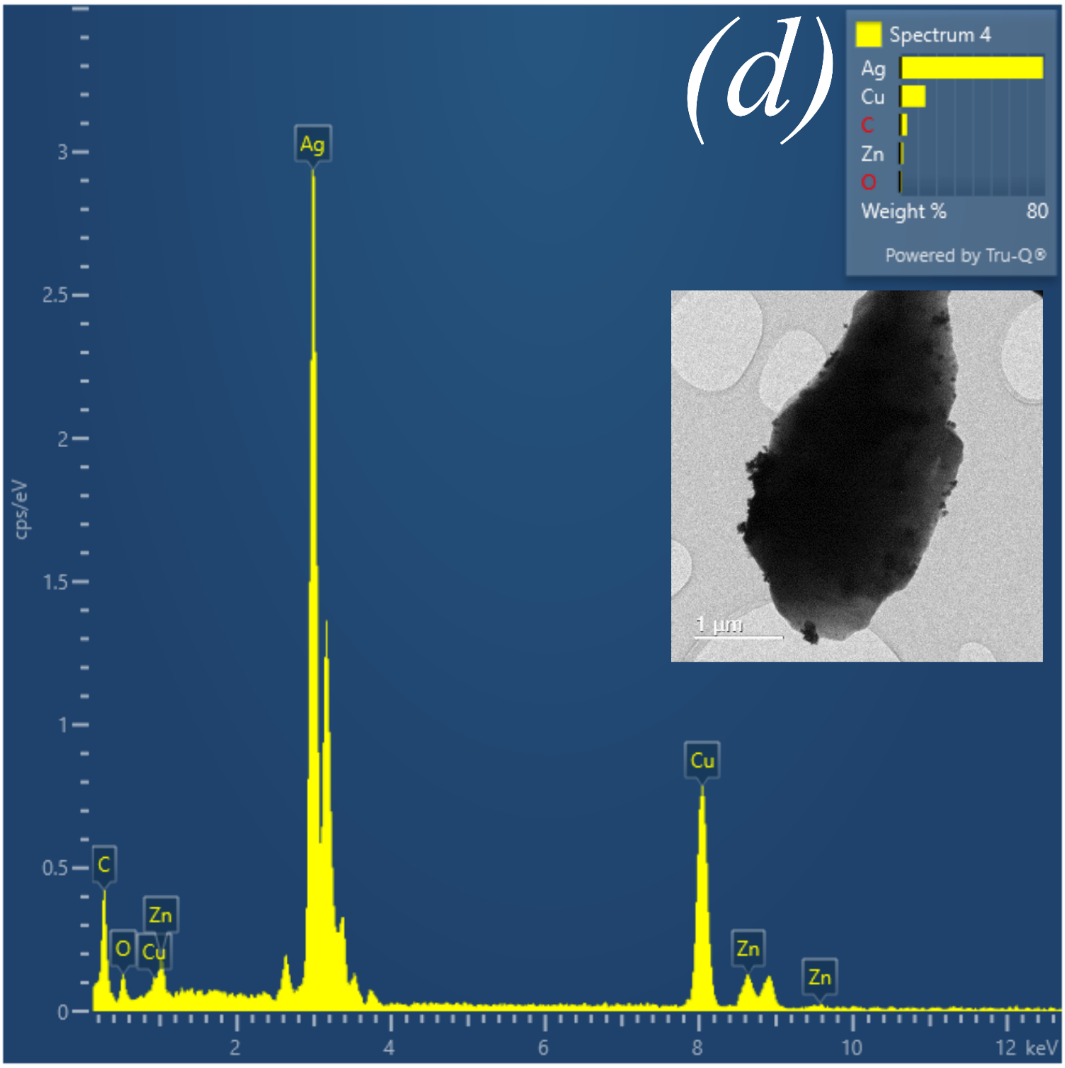
(d)
Spectrum 4
Ag
Cu
C
Zn
O
Weight %
80
Powered by Tru-Q®
1 µm
cps/eV
0
0.5
1
1.5
2
2.5
3
2
4
6
8
10
12 keV
Ag
Cu
C
Zn
O
Cu
Zn
Zn

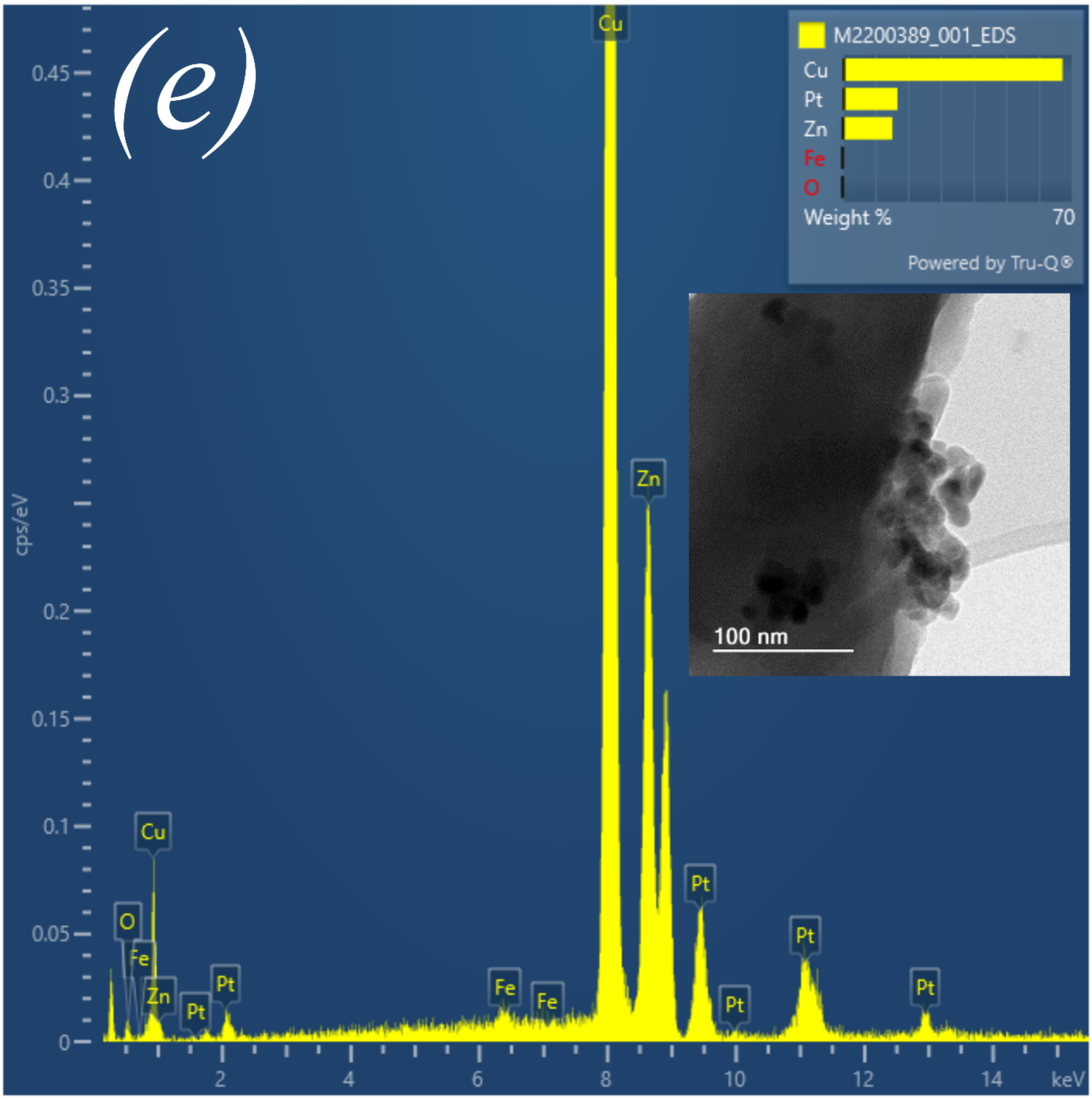

Figure S6: EDS spectra of the MeNPs/Zn-MOF-74 (Me = Ag, Au, Pt) composites in all cases. It was possible to identify the metal presence: (a) AuNPs/Zn-MOF-74 (UVI), (b)AuNPs/Zn-MOF-74 (UVII), (c) AgNPs/Zn-MOF-74 (UVI), (d) AgNPs/Zn-MOF-74 (UVII) and (e) PtNPs/Zn-MOF-74 (UVII).

4. XPS

MeNPs/Zn-MOF-74 (Me = Pt, Au, Ag) powder samples were prepared over carbon stripe and excited with 1486,6 eV (Al Kα) X-ray beam radiation using the charge compensation functionally provided by the thermal Fisher ESCALAB QXi X-ray Photoelectron Spectrometer. Each observed survey spectrum is shown in Figure S7.

5. RAMAN

The assignments of the modes observed in Raman spectra of the *MeNPs/Zn-MOF-74 (Me = Pt, Au, Ag)* are summarized in Table S1 and in Table S2.

Table S1: Assignment of the Raman spectra vibrational modes for Zn-MOF-74 and PtNPs/Zn-MOF-74 composite.

**Zn-MOF-74 (cm^{-1})**	**PtNPs/Zn-MOF-74 (*UVI*) (cm^{-1})**	**PtNPs/Zn-MOF-74 (*UVII*) (cm^{-1})**	**Assignment**
563.43	563.03	560.67	$\beta_s(COO^-)$
820.84	826.06	820.96	$\beta_{as}(COO^-)$
1270.37	1271.83	1270.32	$\nu(C\text{-}O)_{phenolate}$
1415.55	1413.89	1411.91	$\nu_s(COO^-)$
1504.06	1507.84	1506.14	$\nu_{19b}(CC)_{ar}$
1562.29	1565.74	1564.21	$\nu_{as}(COO^-)$
1619.14	1622.34	1620.38	$\nu_{8a}(CC)_{ar}$

Table S2: Assignment of vibrational bands from Raman spectra of Zn-MOF-74 and AuNPs/Zn-MOF-74 composite.

**Zn-MOF-74 (cm^{-1})**	**AuNPs/Zn-MOF-74 (*UVI*) (cm^{-1})**	**AuNPs/Zn-MOF-74 (*UVII*) (cm^{-1})**	**Assignment**
563.43	564.35	-	$\beta_s(COO^-)$
820.84	823.54	822.35	$\beta_{as}(COO^-)$
1270.37	1275.41	1275.14	$\nu(C\text{-}O)_{phenolate}$
1415.55	1415.96	1415.61	$\nu_s(COO^-)$
1504.06	1508.33	1507.39	$\nu_{19b}(CC)_{ar}$
1562.29	1567.04	1575.67	$\nu_{as}(COO^-)$
1619.14	1622.83	1623.69	$\nu_{8a}(CC)_{ar}$

6. HRTEM

Figure S7 shows the size distribution of the NPs obtained from the analysis of the HRTEM images. The distribution of NPs sizes was calculated by analyzing more than 30 HRTEM

images with different magnifications obtained for each MeNPs/MOF-74 (Me = Pt, Au, Ag) composites. NPs were identified using ImageJ software (https://imagej.net/ij/index.html) and fitted to a circular shape. The histogram presented in the manuscript shows the distribution of the diameters fitted in all these HRTEM images. A lognormal distribution was used to calculate the average diameter of the nanoparticles.

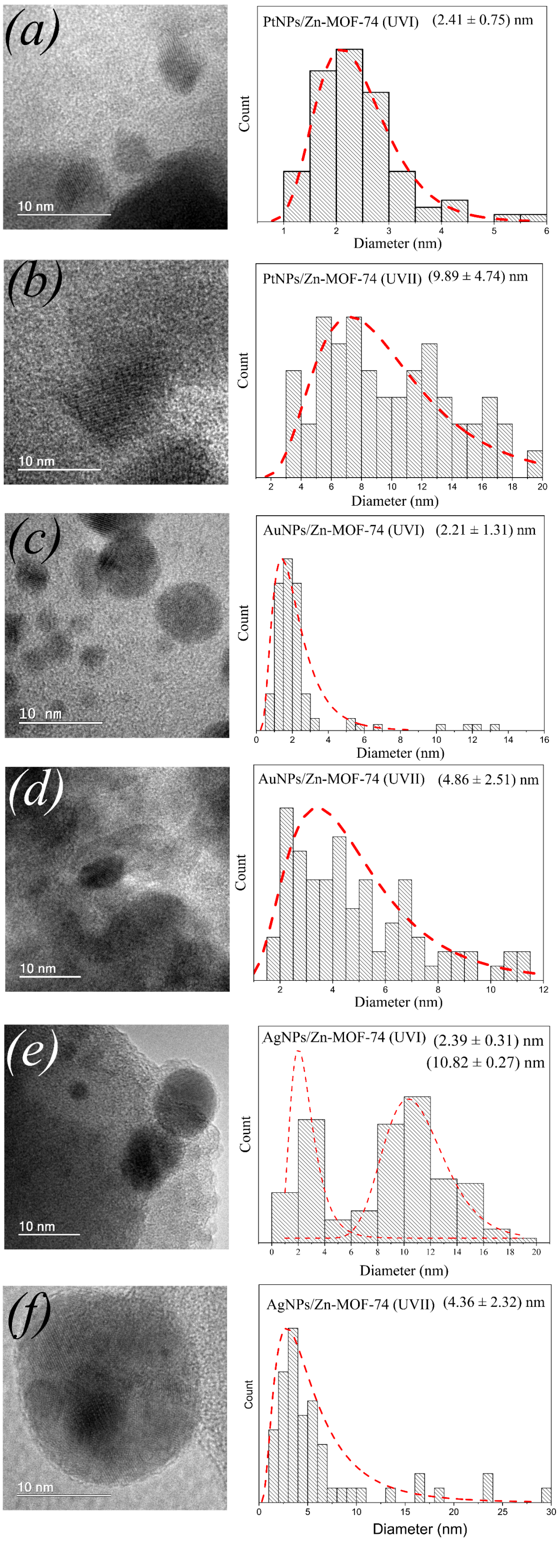

Figure S7: Size distribution of the particles in the (a) PtNPs/Zn-MOF-74 (UVI), (b) PtNPs/Zn-MOF-74 (UVII), (c) AuNPs/Zn-MOF-74 (UVI), (d) AuNPs/Zn-MOF-74 (UVII), (e) AgNPs/Zn-MOF-74 (UVI) and (f) AgNPs/Zn-MOF-74 (UVII).

The high-resolution HRTEM images and the corresponding fast Fourier transform (FFT) patterns of Pt, Au and Ag nanoparticles representative of each sample are shown in Figures S9, S10 and S11. The selected area FFT pattern of the particle revealed the stacking of (111) and (200) planes.

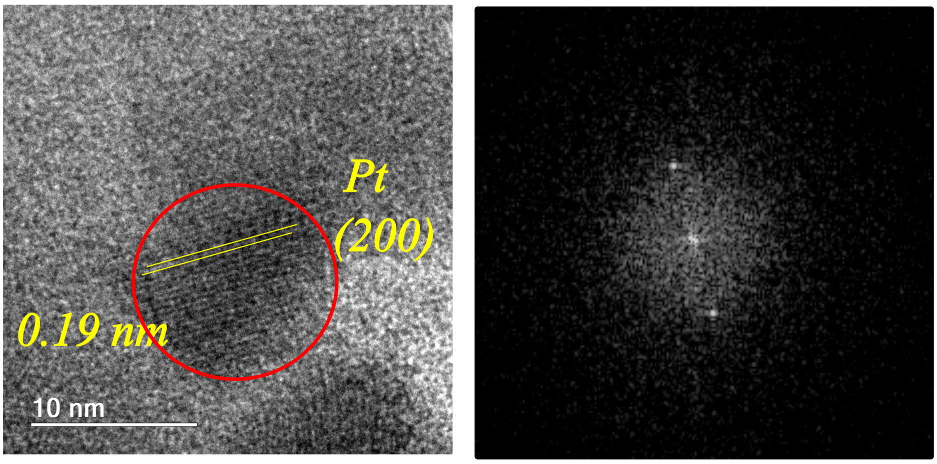

Figure S8: HRTEM image and the FFT pattern of the selected Pt nanoparticle image.

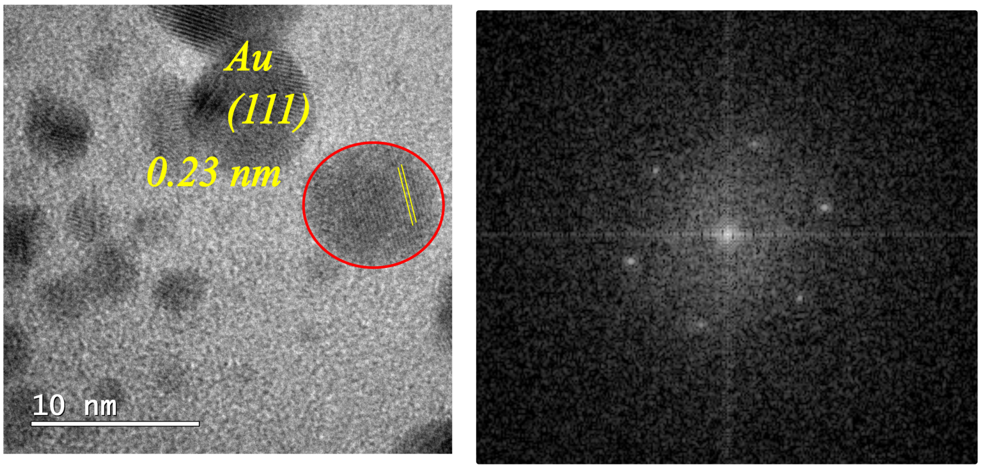

Figure S9: HRTEM image and the FFT pattern of the selected Au nanoparticle image.

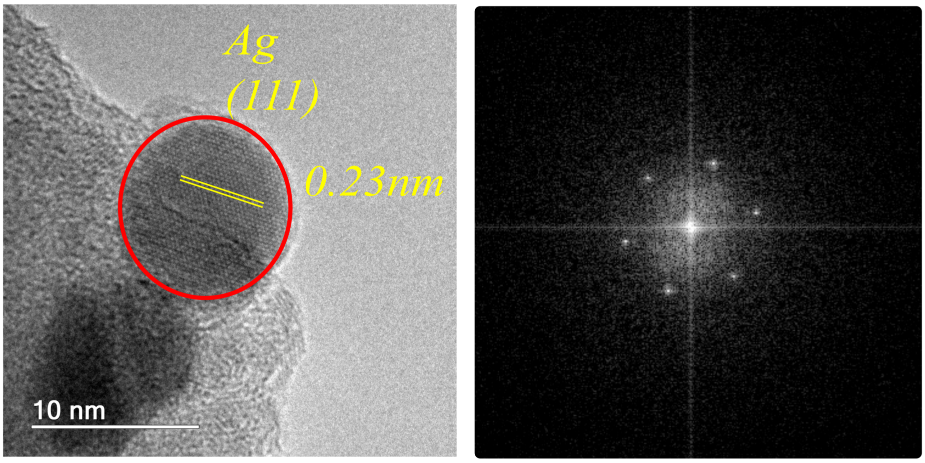

Figure S10: HRTEM image and the FFT pattern s of the selected Ag nanoparticle image.
